# Impact of the duration of farmland restoration on plant communities and soil carbon-nitrogen-phosphorus stoichiometry in the Southern Foothills of the Greater Khingan Mountains, Inner Mongolia

**DOI:** 10.1371/journal.pone.0345060

**Published:** 2026-03-20

**Authors:** Yan Sheng, Fucang Qin, Yongjie Yue, Long Hai, Long Li, Xiaoyu Dong, Danlu Tao, Rong He, Kai Zhao

**Affiliations:** 1 College of Desert Control Science And Engineering, Inner Mongolia Agricultural University, Hohhot, China; 2 Inner Mongolia Academy of Forestry Sciences, Hohhot, China; 3 College of Forestry, Inner Mongolia Agricultural University, Hohhot, China; Shandong University, CHINA

## Abstract

The southern foothills of the Greater Khingan Mountains in Inner Mongolia are one of the 14 contiguous destitute areas in China. The ecological environment in the region is harsh. The implementation of the Grain-for-Green Project is crucial to the restoration and protection of local forest ecosystems. However, the project has some deficiencies in terms of plant communities and soil stoichiometric characteristics. In this study, the southern slope of Daxing’anling Forest in Inner Mongolia was used as the research area, and 1 year, 3 years, 5 years, 10 years, 20 years, abandoned land, and farmland after the restoration were comprehensively selected. The purpose of this study was to analyze the dynamic changes in soil stoichiometric characteristics and plant diversity during ecological restoration of this area, explore the influence of soil stoichiometric characteristics on plant diversity. The following results were obtained: (1) In terms of plant communities, plant species increased first and then decreased after returning farmland. In the early stage, pioneer herbs increased, and some species were eliminated due to competition and other factors in the later stage. Shrubs and trees appeared late, and herbs had strong adaptability. (2) In terms of soil stoichiometry, the contents of soil organic carbon (SOC) and total nitrogen (TN) were low in the early stage of returning farmland. With the increase in years, SOC increased first and then decreased, and TN increased. The changes in each soil layer were affected by many factors and differed from those of the control. The total phosphorus (TP) content fluctuated, and the ratios of C/N, C/P, and N/P had different trends in varying soil layers with returning farmland and vegetation restoration. (3) In terms of the relationship between vegetation and soil nutrients, SOC was positively correlated with TN, TN was positively correlated with the evenness index, and TP was negatively correlated with some vegetation indexes. This work has important guiding significance for improving soil fertility and plant growth in returning farmland to forest. This work is helpful to realize ecological environment protection and sustainable agricultural development.

## 1. Introduction

Carbon (C), nitrogen (N), and phosphorus (P) in soil are essential nutrients for plant growth and development [1]. Their ecological stoichiometric characteristics not only directly affect the mineralization of soil organic matter but also indirectly affect the physical and chemical properties of soil, which are important indicators reflecting soil quality and fertility [2,3]. The stoichiometric ratio of C:N:P is closely related to plant growth, species diversity, community structure and its dynamic changes, and ecosystem processes [4,5]. Among them, soil organic carbon (SOC), soil total nitrogen (TN), and soil total phosphorus (TP) are usually used to represent the overall nutrient level of soil [6]. SOC:TN (C:N) and SOC:TP (C:P) can reflect the quality and decomposition rate of organic matter [7], whereas TN:TP (N: P) can reflect nutrient limitation during plant growth [8]. Therefore, studying the ecological stoichiometric characteristics of soil C, N, and P is of great significance for understanding the biogeochemical cycle of ecosystems, improving the ecological benefits of ecosystems, and optimizing soil management strategies.

The Greater Khingan Mountains in Inner Mongolia are located in northeast China, and they are the main distribution area of cold temperate coniferous forests in northern China, covering important ecological areas such as the upper reaches of the Nenjiang River and the Erguna River Basin [**9**]. The southern foothills of the Greater Khingan Mountains are located in the hilly area of the southern end of the Greater Khingan Mountains. They are one of the 14 concentrated contiguous destitute areas in China [**10**]. The ecological environment in this area is relatively poor, characterized by serious soil erosion and frequent natural disasters [**11**]. The implementation of the Grain-for-Green Project will help restore and protect the forest ecosystem in the Greater Khingan Mountains; improve the regional ecological environment; and effectively enhance the soil structure, increase biodiversity, reduce soil erosion, and prevent land degradation [12,13]. Therefore, the ecological breakthrough in the southern foothills of the Greater Khingan Mountains lies in the implementation of the project of returning farmland to forest. Although returning farmland to forest has brought many ecological benefits, it still faces some challenges in the implementation process. In terms of plant community and soil stoichiometric characteristics, there are still some shortcomings in returning farmland to forest, including single plant community structure, unbalanced soil nutrients, and insufficient long-term monitoring [14–16]. Therefore, the measures of returning farmland to forest must be further studied and optimized to improve the ecological environment quality in the southern foothills of the Greater Khingan Mountains. By strengthening the diversity of plant communities, balancing soil nutrients, and establishing long-term monitoring mechanisms, the ecological benefits of returning farmland to forests can be better utilized, providing a scientific basis for the sustainable development of the regional ecological environment.

Previous studies have reached a consistent conclusion regarding the variation in plant diversity in the process of returning farmland to forest restoration, that is, in the early stage of restoration, plant diversity gradually increases with the increase in restoration years [17]. However, in the later stage of restoration, when the dominant species in the community are prominent, plant diversity will decrease [18]. However, the magnitude and trajectory of this change can vary significantly across regions due to differences in climate, soil type, and initial species pool. Furthermore, a key point of discussion centers on how plant diversity in restored (abandoned) land compares to that in actively managed, non-abandoned farmland. Some studies suggest that abandonment promotes diversity by allowing natural succession, while others indicate that managed farmland can maintain a different but potentially species-rich community depending on practices. The maximum value of species diversity in the community may appear in the middle and late stages of restoration [**19**]. With the change in restoration years of returning farmland to forest, the nutrient cycles of the plant–soil system and its relationship with plant diversity are unclear. Many scholars have studied land use change, climate change, and vegetation succession [20–22], and they found that vegetation succession and soil stoichiometric characteristics are important bases for judging the structure and function recovery of an ecosystem [23–25]. Plant community diversity characteristics such as species richness, Shannon–Wiener diversity index and Simpson dominance can measure vegetation succession and plant community function. Moreover, the aboveground parts of plants usually have a certain correlation with the soil nutrient content [**26**]. Some studies have shown that soil stoichiometry is a limiting factor for plant growth and development under certain conditions [**27**]. At present, the relationship between restoration years of abandoned farmland and vegetation succession and soil stoichiometric characteristics in Daxing’anling Mountains remains unclear. Studying the effects of plant–soil C, N, and P and their stoichiometric ratios on plant diversity in different duration of restoration is helpful to understand the mutual feedback mechanism between plant and soil nutrients and plant diversity, and it has important guiding significance for reflecting the improvement of vegetation restoration and soil nutrient status in this area.

At present, researchers are paying increasing attention to soil stoichiometry (C:N:P) to explore the biogeochemical cycle [**28**]. However, for large ecologically fragile areas, the spatial patterns, succession patterns, impacts, and driving factors of this stoichiometry are still poorly understood. Research on soil ecological stoichiometry in the Greater Khingan Mountains mainly focuses on forest age, vegetation composition, altitude, and stand density [**29**], but few studies have investigated soil stoichiometric characteristics at different succession stages and returning time. By comparing the carbon, nitrogen and phosphorus stoichiometric ratios of plant communities and soils in different restoration time restoration sites with farmland and abandoned land, we can better evaluate the effectiveness of restoration work. Farmland is used as a control of the pre-recovery state, and abandoned land provides a reference for natural recovery. This comparison can help us to determine the extent to which restoration measures improve ecological conditions compared to pre-restoration status and natural restoration processes [30,31]. In this study, 1a, 3a, 5a, 10a, and 20a of returning farmland to forest and abandoned land and farmland after the restoration were selected as control groups (CK1 and CK2) in the southern foot of the Greater Khingan Mountains in Inner Mongolia. The dynamic changes in soil stoichiometric characteristics and plant diversity during the restoration of ecological restoration were analyzed, and the effects of soil stoichiometric characteristics on plant diversity were discussed. The aim of this work was to provide a scientific basis for vegetation restoration and management after returning farmland to forest in the Daxing’anling area. This study is the first to comprehensively investigate and analyze the effects of different years of returning farmland on plant succession and soil nutrient elements, so the results have important guiding significance for improving soil fertility and plant growth in returning farmland to forest. We proposed three hypotheses as follows: 1. If the richness of plant species increases monotonically over time, will the soil stoichiometric characteristics also exhibit a single – linear variation to ensure the stable growth of plants? 2. In the early stage of farmland restoration, if the contents of soil organic carbon and total nitrogen are relatively high, will this cause a change in the succession pattern of the plant community? 3. Considering the intricate relationship between vegetation and soil nutrients, if the fluctuation in the soil total phosphorus content has no significant impact on plant diversity indices, will the correlation between plant diversity and the stability of the soil ecosystem be attenuated during the ecological restoration of farmland?

## 2. Materials and methods

### 2.1. Overview of the study area

The study area is located in the southern foothills of the Greater Khingan Mountains in Inner Mongolia, Inner Mongolia Autonomous Region Hulun Buir City Oroqen Autonomous Banner Dayangshu Town, located in the east longitude 124°14′ to 128°51′ and latitude 49°21′ to 50°13′. The terrain is high in the northwest and low in the southeast, with shallow hills in the northwest and rolling hills in the southeast. The altitude is between 300 and 450 m, and the soil is mainly black soil and dark brown loam. Dayangshu Town is characterized by a temperate continental monsoon climate. The winter is long and cold, the summer is short and warm, and the spring and autumn transition is rapid. The annual average temperature is about −0.8 °C, and the annual average precipitation is 450 mm. The rainfall is mainly concentrated from July to August. The project of returning farmland to forest in Dayangshu Town began in 2000 and was fully launched in 2002. The area of returning farmland to forest is about 2213 hm^2^. The native tree species mainly include *Betula platyphylla*, *Betula davurica*, and *Quercus mongolica*. *Populus przewalskii*, *Larix gmelinii*, and other tree species are commonly used in returning farmland to forest. Common herbaceous plants include *Setaria viridis* and *Eriophorum scheuchzeri*, and shrub species include *Rhododendron simsii* and *Lespedeza bicolor*.

### 2.2. Methods

#### 2.2.1. Arrangement of sample plot.

**Fig 1:**
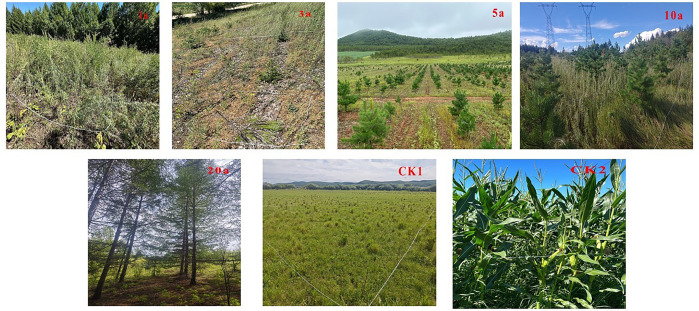
Different years of returning farmland and control plots were photographed.

In July and August of 2023 and 2024, we conducted in-depth field research visits in the study area. Through the comprehensive exploration of local topography, land use history, and other factors, the time of returning farmland in each region was determined. On this basis, the returned farmland plots with similar terrain conditions (visually assessed to have comparable slope position and gradient) and consistent soil types (determined by field observation of topsoil texture and color) were strictly selected as the sample plots. We adopted the space-for-time substitution method. Sample plots representing different durations since farmland return — namely 1 year (1a), 3 years (3a), 5 years (5a), 10 years (10a), and 20 years (20a) — were established. The selection of these specific time points was based on two primary considerations: (1) the actual implementation timeline of the “Grain-for-Green” project in this region, which began in the early 2000s, making ~20 years the longest continuous restoration duration available for study; and (2) the intention to capture key phases of ecological succession, encompassing initial (1-3a), intermediate (5-10a), and longer-term (20a) recovery stages, thereby allowing observation of nonlinear dynamics in soil and vegetation properties. Additionally, two control plots (CKs) were set up: CK1 represents abandoned land undergoing natural restoration after farmland return (i.e., no agricultural activities post-return, allowing natural ecological succession), and CK2 remains as actively managed farmland (with regular cropping and management practices continuing). The core premise of this method is that by carefully selecting plots that are similar in key background conditions (terrain and soil) but differ in the time since farmland return, the observed differences in vegetation and soil properties among these plots can be reasonably attributed to the progression of restoration time, rather than to other confounding environmental factors. The details of the sample plots are shown in Table 1 and Fig 1.

**Table 1:** Sample plot conditions.

Restoration years	Forestry center	Longitude	Latitude	Gradient
1a	Yi li	124.505491	49.686392	sloping land 13°
3a	Ku mo	123.948803	49.742029	sloping land 16°
5a	Da yang shu	124.677219	49.737010	sloping land 18°
10a	Yi li	124.058641	49.589129	sloping land 17°
20a	Wu lu bu tie	124.414649	49.849643	sloping land 15°
CK1 fallow land	Da yang shu	124.380585	49.798158	flattened
CK2 farmland	Yi li	124.102025	49.308670	flattened

Note: In the table, ‘a’ is the abbreviation of ‘year’, representing the time span. For example, ‘1a’ stands for 1 year, ‘3a’ stands for 3 years, and so on.

#### 2.2.2. Sample collection and processing.

In the seven selected types of land, we demarcated three sample plots of 10m × 10m each. In each plot, five 1m × 1m herb quadrats were evenly arranged along the diagonal direction. All plant species names that appeared in the quadrat were identified and recorded. For each plant, the basic indexes such as individual number, height, and coverage were counted. Finally, the relevant indexes of plant communities were calculated based on these basic data.

Given that the study area is located in the rocky mountain area, the soil layer is relatively thin, so our soil sampling depth was set at 0–60 cm. Sampling was performed every 20 cm. When sampling each layer, three different positions were evenly selected in the plot for repeated sampling, and the three samples in the same layer were fully and evenly mixed to form a mixed soil sample representing the layer. The collected soil samples were brought back to the laboratory for subsequent determination. SOC was determined by potassium dichromate method, TN was determined by semi-micro Kjeldahl method, and TP was determined by sulfuric acid–perchloric acid digestion method [**32**].

#### 2.2.3. Data analysis.

Important value (Pi), species richness index (S), Simpson dominance index (D), Shannon–Wiener diversity index (H’), Pielou evenness index (E), and Margalef richness index (R) were calculated using the following formulas [33,34]:

(1) Important value (Pi)

$$P_{i}=n_{i}/N$$

Where *n*_*i*_ represents the number of individuals of plant species *i* and *N* represents the total number of individuals of all plant species in one particular quadrat, respectively.

(2) Species richness index (S):

S=s

(3) Simpson dominance index (D):

$$\text{D}=\text{1}-\sum {\text{Pi}}^{\text{2}}$$

(4) Shannon-Wiener diversity index (H’):

$${\text{H}}^{\prime }=-\sum \text{Pi}\cdot \text{ln}\text{Pi}$$

(5) Pielou evenness index (E):

E=(−∑Pi·lnPi)/lns

(6) Margalef richness index (R):

R=(s−1)/lnT

In the formulas, *s* is the number of species of plant communities; *Pi* is the relative abundance of species *i* over the total abundance; i = 1,2,3...... n; and *t* is the total number of individuals of all species in the quadrat.

#### 2.2.4. Data processing.

After using Excel 2019 to conduct preliminary statistical collation of all data, the important values of vegetation and diversity index were calculated. SPSS26.0 was used to perform normality test, homogeneity of variance test, one-way ANOVA, and Pearson correlation analysis on each dataset. All data in the chart are presented as the mean ± standard deviation, and Origin 2021 was used for graphical representation.

## 3. Results

### 3.1. Species composition and dominance of different duration of restoration of returning farmland

In the plant communities with different duration of restoration, a total of 29 species were investigated, which belonged to 11 families and 24 genera (Table 2). Among them, Asteraceae, Poaceae, and Leguminosae have more plant species than the other families, with 11, 6, and 3 species, respectively. The plants of these three families account for 70.0% of the total species. From the perspective of genera, *Stipa*, *Lespedeza*, and *Suaeda* each contain two species; *Artemisia* has three species; and there are 20 species of single genera, accounting for 70.0% of the total species. According to the life form, these plants can be divided into 3 shrubs, 13 perennial herbs, 12 annual herbs, and 1 small tree, accounting for 10.3%, 44.8%, 41.4%, and 3.4%, respectively.

**Table 2:** Vegetation community composition at different years of fallow.

Species	Families	Life from	Years of fallow restoration
*Erigeron bonariensis* L.	Asteraceae	annual herb	1a、3a、CK1
*Tribulus terrestris* L.	Zygophyllaceae	annual herb	1a、3a、CK1
*Taraxacum mongolicum* Hand.-Mazz.	Asteraceae	herbaceous perennial	1a、3a、5a、CK1
*Setaria viridis* P. Beauv.	Poaceae	annual herb	1a、3a、5a、CK1
*Artemisia sieversiana* Ehrhart ex Willd.	Asteraceae	annual herb	1a、3a、5a、10a、CK1
*Erigeron canadensis* L.	Asteraceae	annual herb	1a、3a、CK1
*Artemisia lavandulifolia* DC.	Asteraceae	herbaceous perennial	3a、5a、10a、CK1
*Geum aleppicum* Jacq.	Rosaceae	herbaceous perennial	3a、5a、CK1
*Vicia sepium* L.	Fabaceae	herbaceous perennial	3a
*Artemisia argyi* H. Lév. & Vaniot	Asteraceae	herbaceous perennial	3a、5a
*Aster altaicus* Willd.	Asteraceae	herbaceous perennial	3a、5a、10a、CK1
*Atractylodes lancea* DC.	Asteraceae	herbaceous perennial	5a
*Leymus chinensis* Tzvelev.	Poaceae	herbaceous perennial	5a、CK1
*Pogonatherum paniceum* Hack.	Poaceae	herbaceous perennial	5a、10a
*Lespedeza bicolor* Turcz.	Fabaceae	shrub	10a、20a
*Corylus heterophylla* Fisch. ex Trautv.	Betulaceae	shrub	10a、20a
*Carex lanceolata* Boott.	Cyperaceae	annual herb	5a
*Inula japonica* Thunb.	Asteraceae	herbaceous perennial	5a、CK1
*Oenothera biennis* L.	Onagraceae	annual herb	5a
*Stipa capillata* L.	Poaceae	herbaceous perennial	3a、5a、CK1
*Echinochloa crus-galli* P. Beauv.	Poaceae	annual herb	3a、5a
*Suaeda stellatiflora* G. L. Chu	Amaranthaceae	annual herb	3a、CK1
*Tamarix chinensis* Lour.	Tamaricaceae	microphanerophytes	20a
*Lespedeza cuneata* G. Don.	Fabaceae	shrub	10a
*Sonchus oleraceus* L.	Asteraceae	annual herb	3a、5a
*Suaeda glauca* Bunge.	Amaranthaceae	annual herb	5a、10a、20a
*Ixeris polycephala* Cass. ex DC.	Asteraceae	annual herb	5a
*Argentina anserina* Rydb.	Rosaceae	herbaceous perennial	10a
*Stipa bungeana* Trin.	Poaceae	herbaceous perennial	10a、CK1

We observed significant differences in the number of plant community species under different years of returning farmland, and the number of plant community species showed a fluctuating trend with the increase in restoration years. Specifically, the number of species in 1a, 3a, 5a, 10a, 20a, and CK1 were 6, 15, 18, 10, 4, and 14, respectively. Shrubs only appeared in 10a and 20a, and trees only appeared in 20a, whereas 1-year-old and perennial herbs were distributed in all years of returning farmland. *A. sieversiana*, *T. mongolicum*, and *A. lavandulifolia* appeared more frequently than the other species in different years of returning farmland. *A. lancea*, *C. lanceolata*, and *I. polycephala* only appeared in 5 years of returning farmland. *L. cuneata* and *A. anserina* only appeared in 10 years of returning farmland.

The dominant plants of 1a and 3a were *T. mongolicum*. The dominant plant of 5a was *A. lavandulifolia*. The dominant plant of 10a and 20a was *L. bicolor*. The dominant plant of CK1 was *A. sieversiana*. The dominant plant of CK2 was *Z. mays*. The important values of dominant plants in different duration of restoration are listed in Table 3.

**Table 3:** Species importance values of vegetation communities at different duration of restoration.

Years of fallow restoration	Species	Importance value
1a	*T. mongolicum* Hand.-Mazz.	0.2541
	*S. viridis* P. Beauv.	0.2236
	*A. sieversiana* Ehrhart ex Willd.	0.1672
3a	*T. mongolicum* Hand.-Mazz.	0.3784
	*A. lavandulifolia* DC.	0.2130
	*A. sieversiana* Ehrhart ex Willd.	0.1965
5a	*A. lavandulifolia* DC.	0.4658
	*A. altaicus* Willd.	0.2574
	*L. chinensis* Tzvelev.	0.1840
10a	*L. bicolor* Turcz.	0.4110
	*A. lavandulifolia* DC.	0.2685
	*P. paniceum* Hack.	0.1361
20a	*L. bicolor* Turcz.	0.6000
	*C. heterophylla* Fisch. ex Trautv.	0.2000
CK1	*A. sieversiana* Ehrhart ex Willd.	0.1550
	*S. viridis* P. Beauv.	0.1283
	*S. capillata* L.	0.1037
CK2	*Zea mays* L.	0.4500
	*Glycine max* L.	0.3500

### 3.2. Analysis of plant community diversity in different duration of restoration

Plant community diversity refers to the rich diversity of plant communities in composition, structure, function, and dynamics, so it plays an important role in the field of community ecology. In addition, the higher the vegetation species richness and the more complex the community structure, the more perfect the forest ecosystem function. In Table 4, the Margalef richness index reached the highest value at 5a and the lowest at 1a. The peak values of Shannon–Wiener diversity index and Simpson dominance index appeared in CK1, and the lowest value appeared at 20a. The Pielou evenness index showed a trend of increasing first and then decreasing during the period of 1–20 years of returning farmland. Its maximum value was observed at 5 years of returning farmland, slowly rebounded during the period of 10–20 years of returning farmland, and stabilized. The Pielou evenness index of CK2 was the highest, whereas that of 1a was the lowest. The above results revealed that the habitat stability of CK1 in Daxing’anling Mountains was the highest under different years of returning farmland. The fluctuation of community characteristics under different years of returning farmland indicated that the habitat was unstable during the process of returning farmland.

**Table 4:** Changes in vegetation community diversity among different years of fallow restoration.

Years of fallow restoration	MR	SWD	SD	PE
1a	0.33 ± 0.20^c^	0.73 ± 0.11^ab^	0.89 ± 0.35^a^	0.14 ± 0.10^ab^
3a	1.50 ± 0.52^ab^	1.66 ± 0.32^ab^	2.25 ± 1.20^a^	0.35 ± 0.15^ab^
5a	3.53 ± 1.20^a^	2.72 ± 0.48^ab^	0.31 ± 0.12^a^	0.42 ± 0.21^ab^
10a	1.67 ± 0.83^ab^	1.43 ± 0.46 ^ab^	1.31 ± 0.52^a^	0.27 ± 0.17^ab^
20a	1.50 ± 0.26^ab^	0.57 ± 0.35^ab^	0.17 ± 0.10^a^	0.35 ± 0.20^ab^
CK1	2.85 ± 0.90^ab^	3.50 ± 0.59^a^	2.70 ± 0.55^a^	0.52 ± 0.32^ab^
CK2	2.51 ± 0.33^ab^	1.22 ± 0.25^a^	2.15 ± 0.48^a^	0.90 ± 0.25^b^

Notes: lowercase letters indicate that the same indicator was significantly different (P < 0.05) between different years of fallow restoration, MR is the species richness index; SD is Simpson dominance index; SWD is Shannon–Wiener diversity index; PE is Pielou evenness index; and MR is Margalef richness index.

### 3.3. Soil stoichiometric characteristics of different years of returning farmland

**Fig 2:**
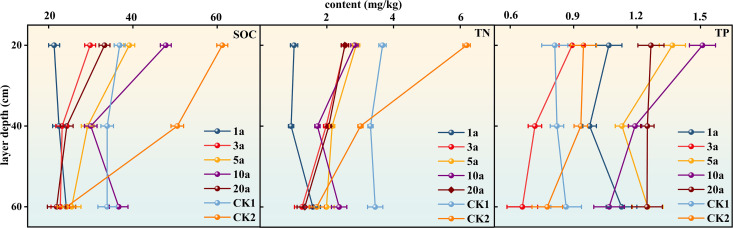
Variation in soil SOC, TN, and TP in different years of returning farmland.

In Fig 2, In terms of SOC, the SOC content in the 0–20 cm soil layer of farmland was the highest. With the increase in the number of years of returning farmland, the SOC content initially increased and then decreased, reaching a high level after 10 years of returning farmland. By contrast, the SOC content of abandoned land was significantly lower than that of farmland but higher than that of land in the early stage of returning farmland. In the 20–40 cm soil layer, the SOC content of farmland remained at a high level, whereas the SOC content of abandoned land was lower than that of farmland. The SOC content of land with different years of returning farmland also showed a trend of increasing first and then decreasing, and the overall content was lower than that of farmland. In the 40–60 cm soil layer, the SOC content of abandoned land was higher than that of farmland. The SOC content of land with different years of returning farmland did not change significantly. The SOC content of some years (such as 10 years of returning farmland) was higher than that of farmland, but the content of 20 years of returning farmland was low.

In terms of TN content, the TN content in the 0–20 cm soil layer was lower in the early stage of returning farmland. In the 20–40 cm soil layer, the TN content gradually increased in 1a to 20a. In the 40–60 cm soil layer, the TN content decreased first and then increased. Compared with the control groups CK1 and CK2, the TN content of different soil layers also showed differences in different years of returning farmland. In the 0–20 cm soil layer, the TN content of CK1 was higher than that of returning farmland in some years, whereas in the 20–40 cm soil layer, the TN content of returning farmland for 20 years was higher than that of CK2.

In terms of TP content, the TP content in the 0–20 cm soil layer fluctuated, increasing from 1 a to 10 a, and then decreasing. In the 20–40 cm soil layer, the TP content also fluctuated, rising from 1 a to 10 a, and then changed. In the 40–60 cm soil layer, TP content also showed a fluctuating state. Compared with the control group CK1 and CK2, the TP content of different soil layers also showed differences in different years of returning farmland. For example, in the 0–20 cm soil layer, the TP content was higher than that of CK1 and CK2.

**Fig 3:**
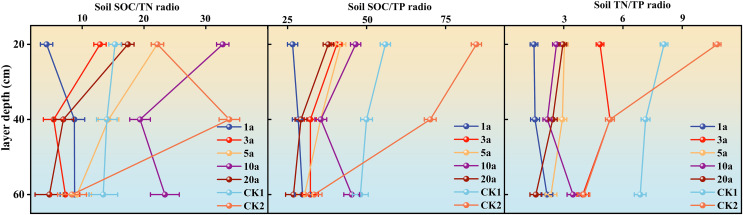
Variation in soil C/N, C/P, and N/P in different years of returning farmland.

We calculated the C/N, C/P and N/P of different years of returning farmland and two controls (Fig 3). The results showed that the change trend of C/N was not completely consistent under different soil depths. In general, some soil layers showed a trend of increasing first and then decreasing, and there were significant differences between different soil layers. In the 0–20 cm soil layer: C/N increased from 1 a to 10 a, reached a higher value, and then decreased in 20 a. In the 20–40 cm soil layer: C/N increased from 1 a to 10 a, reached a higher value, and then decreased significantly in 20 a. In the 40–60 cm soil layer: C/N increased from 1 a to 10 a, reached a higher value, and then decreased in 20 a. Compared with the control: in the 0–20 cm soil layer: the C/N of 10 a was higher than that of CK1 and CK2; there were different degrees of difference in C/N between other years of returning farmland and the control. In the 20–40 cm soil layer: the C/N of 10 years of returning farmland was higher than that of CK1, which was different from that of CK2, and the C/N of different years of returning farmland was different from that of CK. In the 40–60 cm soil layer: the C/N of each returning year and CK1 and CK2 also had different levels of content.

C/P also showed the characteristics of fluctuation at different soil depths, and the variation range and trend of different soil layers were different. In the 0–20 cm soil layer: C/P increased from 1 a to 10 a, and then decreased in 20a. In the 20–40 cm soil layer: C/P began to rise from 1 a, reached a higher value in 10 a, and then decreased in 20 a. In the 40–60 cm soil layer: C/P began to rise from 1 a, reached a higher value in 10 a, and then decreased in 20a. Compared with the control: in the 0–20 cm soil layer: the C/P of 10 years of returning farmland was lower than that of CK1 and CK2, and the C/P of other years of returning farmland was different from that of the control. In the 20–40 cm soil layer: the C/P ratio of each returning year to CK1 and CK2 was diverse, with high and low. In the 40–60 cm soil layer: the C/P ratio of CK1 and CK2 showed different levels with different years of returning farmland.

### 3.4. Correlation analysis of vegetation and soil nutrients in different duration of restoration of returning farmland

Pearson correlation analysis between plant community characteristics and soil ecological stoichiometry characteristics at different restoration durations showed that SOC and TN were significantly positively correlated (0.853*, *P* < 0.05). SOC was also positively correlated with MR, SWD, PE, and SD, particularly with PE (0.793*, *P* < 0.05). These positive correlations suggest a potential link where higher SOC levels are associated with greater vegetation richness, diversity, and evenness. TN was negatively correlated with TP (−0.37), but the association was not significant. TN was positively correlated with MR, SWD, PE, and SD, particularly with PE (0.881**, *P* < 0.01), indicating that TN, similar to SOC, showed a positive association with plant diversity indices. The correlation between TP and MR was very weak (correlation coefficient was 0.002). TP was negatively correlated with SWD, SD, and PE, particularly with SD (−0.882**, *P* < 0.01). Thus, the observed negative correlations imply that increased TP content may not be associated with higher plant diversity. MR was significantly positively correlated with SWD (0.761*, *P* < 0.05) and showed positive but weak correlations with SD and PE, indicating that the richness index is related to other diversity indicators. SWD was positively correlated with SD and PE, and SD was also positively correlated with PE, suggesting a certain synergistic relationship among the diversity, dominance, and evenness indices (Table 5).

**Table 5:** Correlation analysis of vegetation and soil in different duration of restoration of returning farmland.

	years	SOC	TN	TP	MR	SWD	SD	PE
years	1							
SOC	0.388	1						
TN	0.775*	0.853*	1					
TP	−0.574	−0.086	−0.37	1				
MR	0.723	0.544	0.715	0.002	1			
SWD	0.677	0.233	0.536	−0.281	0.761*	1		
SD	0.529	0.412	0.601	−0.882**	0.14	0.431	1	
PE	0.746	0.793*	0.881**	−0.402	0.594	0.222	0.466	1

Note: In the table, * is a significant correlation between the two (*P* < 0.05); ** is the extremely significant correlation between the two (*P* < 0.01); years is the restoration period of returning farmland, the unit is a; SOC is soil organic carbon content, the unit is mg/kg; TN is soil total nitrogen content, unit is mg/kg; and TP is the total phosphorus content of soil, the unit is mg/kg.

## 4. Discussion

### 4.1. Changes in plant community characteristics and dominance in different duration of restoration of returning farmland

In the early stage of returning farmland (1–3a), plant species richness began to increase gradually. As a result of the cessation of agricultural disturbance, seeds of pioneer plants that were previously suppressed began to germinate. These pioneers, often annual herbs with high adaptability such as E. bonariensis and T. terrestris, can colonize the new environment rapidly, increasing the number of species from 6 at 1a to 15 at 3a. By 3–5a, more plant species, including perennial herbs, appeared. The improvement in soil conditions at this stage—characterized by a looser structure and enhanced fertility—provided a more suitable environment for plant establishment and growth [**35**], supporting a further increase in species number from 15 to 18. From 5a to 20a, the number of plant species showed a declining trend. As vegetation developed, competition intensified, and less competitive species were gradually excluded [**36**]. Furthermore, the long-term natural succession process tends toward a stable community structure, leading to the disappearance of some transitional species present in earlier stages. For instance, the species number decreased to 4 by 20a. This pattern aligns with classic succession models and theory, which highlight the increasing role of competition and species exclusion over time. During succession, pioneer species with strong competitive ability for light, nutrients, and space often become dominant. Through mechanisms such as resource pre-emption, allelopathy, and microenvironment modification, they can inhibit or exclude other species, resulting in reduced species diversity and a more stable, dominant community structure. For example, studies have shown that competitive exclusion of herbaceous plants by woody plants during secondary succession is a key driver of community structural change [**37**].

Shrubs and trees appear only when the recovery period is long (shrubs appeared in 10a and 20a, and trees only appeared in 20a), because they have a long growth cycle and relatively high environmental requirements [**38**]. In the early stage of returning farmland, soil conditions, light, and other environmental factors may not be suitable for their growth. With the gradual restoration of vegetation, the soil layer is thickened, fertility is increased, the water conservation ability is improved, and a certain shade condition is formed, which creates favorable conditions for the growth of shrubs and trees. One-year-old and perennial herbs appeared in all the years of returning farmland, because they had strong adaptability [**39**]. One-year-old herbs can complete their life history in a short time period and grow and reproduce rapidly using short-term suitable environmental conditions. For example, they can grow 1 year after the land has just been returned to farmland. Perennial herbs have strong resource acquisition and storage capacity, can continue to grow in a changing environment, and can occupy a certain position in the community at different stages. Moreover, invasive species may have an important impact on ecosystem restoration. For example, invasive species may alter the physical and chemical properties of the soil, which in turn affects the growth of native plants [**40**]. In addition, invasive species may also inhibit the growth of native plants by competing with native species for resources such as light, water and nutrients [**41**]. In some cases, invasive species may even change the structure and function of ecosystems, with long-term effects on vegetation restoration processes [**42**].

Plants with frequent occurrences across the restoration chronosequence (such as *A. sieversiana*, *T. mongolicum*, and *A. lavandulifolia*) exhibited extensive adaptability and effective dispersal. They maintained a high frequency of occurrence, likely due to their ability to tolerate a range of environmental conditions present during different restoration stages, coupled with diverse seed dispersal modes (e.g., wind, animal). In contrast, plants that appeared only in a specific restoration year (e.g., *A. lancea*, *C. lanceolata*, and *I. polycephala* only at 5a; *L. cuneata* and *A. anserina* only at 10a) appeared to have more specific environmental requirements. For instance, the emergence of *A. lancea* at 5a may indicate that the soil moisture, temperature, and nutrient conditions at that specific time were particularly suitable for its germination and establishment. Studies have shown that soil moisture and temperature are key factors regulating seed germination and seedling growth. The early restoration stage (e.g., 5a) might provide the moderately high soil moisture and temperature that create an ideal microenvironment for *A. lancea* [**43**]. Similarly, the appearance of *L. cuneata* and *A. anserina* at 10a could be linked to the attainment of specific soil nutrient thresholds or structural improvements favorable to these species. With increasing restoration time, the gradual accumulation of soil organic matter and improvement in soil structure [**44**] may create niches for such species with particular nutrient demands (e.g., for nitrogen or phosphorus).

### 4.2. Changes in soil stoichiometric characteristics in different duration of restoration

In the early stage of farmland return (1a), the SOC content was low. This can be attributed to the historical disturbance from intensive agricultural practices (e.g., tillage), which destroyed soil aggregates and accelerated the decomposition of native organic carbon. Concurrently, initial vegetation recovery was limited, resulting in minimal fresh organic carbon input from plant residues [**45**]. In contrast, the highest SOC content in the 0–20 cm layer of active farmland (CK2) is primarily due to long-term agricultural management, including crop root exudates, stubble return, and potential organic amendments (e.g., manure or straw incorporation).With increasing restoration time (from 1a to 10a), SOC content generally increased. This trend aligns with progressive vegetation recovery, which enhances plant cover, species richness, and biomass. Greater inputs from root exudates, dead roots, and aboveground litter collectively promoted SOC accumulation.However, after 10 years of restoration, SOC content showed a declining trend. It is important to clarify that this study did not directly measure microbial biomass or enzyme activities. The following interpretation is therefore based on established ecological mechanisms reported in the literature. The later-phase decrease in SOC may be explained by several integrated factors: (1) the establishment of a more stable plant community with potentially slower biomass accumulation rates; (2) the development toward a more complex soil microbial community (as commonly reported in successional studies), which could enhance the mineralization of organic matter, potentially leading to a new equilibrium between carbon input and microbial decomposition; and (3) potential SOC loss through processes like leaching. Notably, the SOC content in long-term abandoned land (CK1) was lower than in active farmland but higher than in early-stage restored plots, reflecting a moderate level of natural carbon input without agricultural disturbance or fertilization.Interestingly, in the 40–60 cm soil layer, the abandoned land exhibited higher SOC content than the farmland. This pattern may result from deeper root growth and turnover in the naturally vegetated abandoned site, promoting subsoil carbon accumulation, whereas agricultural carbon inputs are typically concentrated in the surface layer.

The TN content in the 0–20 cm soil layer was low at the initial stage of returning to farmland. When returning to farmland for 1a, a large amount of nitrogen was taken away due to crop harvest. Long-term tillage causes nitrogen leaching loss and ammonia volatilization, and the initial vegetation recovery was slow, biological nitrogen fixation was weak, and the soil nitrogen input was small. It was mainly maintained by the original nitrogen mineralization and had difficulty in meeting demand [**46**]. With the increase in the number of years of returning farmland, nitrogen-fixing plants such as legumes grew during vegetation restoration. Through biological nitrogen fixation, soil nitrogen input increased, and the absorption and return of nitrogen by plant roots continued to promote the increase in the TN content. The fluctuation of the TN content in different soil layers is related to the distribution of vegetation roots and microbial activities. For example, the TN content in the 20–40 cm soil layer increased from 1a to 20a; as a result of the growth of roots to the deep layer, nitrogen turnover increased and the microbial mineralization of organic nitrogen was enhanced. The 40–60 cm soil layer first decreased and then increased. In the early stage, given the original nitrogen leaching and underutilization of the root system, it increased with the deepening of the root system and the change in microbial activity. The difference from the control group was due to the different nitrogen input and output pathways and intensities. For example, the TN content of CK1 in the 0–20 cm soil layer was higher than that of returning farmland in some years, and the total nitrogen content in the 20–40 cm soil layer was higher than that of CK2 in 20 years.

The fluctuation of the TP content in each soil layer was due to the complex transformation and migration of P element in soil. In the early stage of returning farmland, the original phosphorus availability may change, the TP content of the 0–20 cm soil layer from 1a to 3a was altered, and some inorganic phosphorus was adsorbed and fixed by soil particles, resulting in a decrease in content. With vegetation restoration, plant roots secrete organic acids to activate phosphorus, improve availability, increase plant absorption and return of phosphorus, and affect soil TP content. The TP content increased in 5–10 years of returning farmland, and phosphorus leaching loss was weaker than nitrogen. Different vegetation had varying phosphorus demand and utilization efficiency, and the soil TP content fluctuated in vegetation succession. The difference from the control group was due to the different sources and destinations of phosphorus. For example, the total phosphorus content of the 0–20 cm soil layer was higher than that of CK1 and CK2.

The change trend of C/N under different soil depths was inconsistent, and some soil layers increased first and then decreased. In the early stage of returning farmland, vegetation recovery was slow, soil nitrogen was more easily lost or absorbed by plants than SOC, C/N was low in the 0–20 cm soil layer after returning farmland for 1a. With the increase in the number of years of returning farmland, vegetation was lush, the SOC input increased, and the nitrogen input was relatively lagging behind. Given the time conditions required for nitrogen fixation and transformation, C/N increased, and C/N of each soil layer was relatively high at 10 years of returning farmland. In the late stage of returning farmland, the vegetation community was stable, the nitrogen cycle was balanced, the SOC decomposition and input reached a new state, and C/N decreased. The change range and trend of C/P in different soil layers varied, and the change in phosphorus transformation and availability was not synchronized with SOC. In the early stage of returning farmland, the SOC content was low, phosphorus was reduced due to the adsorption effectiveness of soil particles, C/P was low in the 0–20 cm soil layer after returning farmland for 1a. With vegetation restoration, SOC input increased, plant root exudates activated phosphorus, C/P increased, and C/P in each soil layer increased after 10 years of returning farmland. In the late stage of returning farmland, SOC decomposition or leaching loss, as well as phosphorus accumulation and transformation, altered the effectiveness, resulting in a decrease in C/P. The variation pattern of N/P in different soil layers is complex, and the nitrogen and phosphorus cycles are independent of each other [**47**]. In the early stage of returning farmland, nitrogen and phosphorus were affected by the change in soil original state and the initial stage of vegetation restoration. N/P was low in the 0–20 cm soil layer after returning farmland for 1a. With vegetation restoration, biological nitrogen fixation, plant absorption, return of nitrogen and phosphorus, and other processes interacted to affect N/P. For example, when nitrogen input increased relatively fast and phosphorus activation was slow, N/P increased, such as part of the soil layer after 10 years of returning farmland. During the late stage of returning farmland, the nitrogen and phosphorus cycles reached a new balance or the N/P ratio changed due to leaching and microbial activity changes.

### 4.3. Relationship between vegetation and soil nutrients in different duration of restoration

A significant positive correlation exists between SOC and TN, which is mainly due to the close relationship between their sources and cycling processes within soil ecosystems [**48**]. SOC is mainly derived from plant residues, root exudates, and soil microbial biomass, and the input of TN is largely dependent on these organic substances [**49**]. For example, during the decomposition of plant residues, nitrogen-containing organic matter will gradually mineralize and release nitrogen, and soil microorganisms will participate in the transformation and circulation of nitrogen when using organic carbon for metabolic activities. An increase in SOC content enhances energy and carbon availability for soil microorganisms, promotes the mineralization and fixation of organic nitrogen by microorganisms, and increases the TN content. By contrast, the increase in TN content promotes plant growth, thereby enhancing the intake of plant residues and elevating SOC content. The SOC content was positively correlated with the MR, SWD, PE, and SD. SOC is an important energy substance of plants. Rich SOC can support the growth and reproduction of many kinds of microorganisms. Different microorganisms have distinct metabolic functions and occupy various ecological niches. They interact with one another and promote the diversity and richness of plant communities [**50**]. For PE, elevated SOC content may promote a balanced distribution of resource acquisition across various species, mitigating the intensity of resource competition and fostering evenness. The SD indicates a weak positive correlation, suggesting that SOC influences the formation and persistence of dominant species within the community, potentially by altering soil environmental conditions and resource availability.

TN and TP showed a weak negative correlation. This may be due to the mutual restriction of nitrogen and phosphorus cycling in soil. Nitrogen and phosphorus are essential nutrients in plant growth, and a certain proportionate relationship exists between them. When nitrogen is relatively sufficient, plants may preferentially absorb nitrogen, affecting the absorption and utilization efficiency of phosphorus, and vice versa [**51**]. However, this mutual restriction relationship is rather weak, probably because the sources of nitrogen and phosphorus in the soil are diverse. In addition to plant absorption, many processes occur, such as soil mineral weathering, fertilization, biological nitrogen fixation, and organic matter decomposition. These affect their content and effectiveness, so a negative correlation between them is not significant. The effect of TN on plant diversity was similar to that of SOC. TN is the basic element that constitutes important biological macromolecules such as proteins and nucleic acids in organisms. An increase in TN content can promote plant growth [**52**]. The significant positive correlation between TN and PE may arise from the reasonable supply of TN, which helps different biological species to optimizer resources in the community and reduce the competitive imbalance caused by nitrogen deficiency, maintaining a high level of evenness.

The correlation between TP and MR was very weak, indicating that TP played a relatively small role in directly affecting plant richness. However, TP was negatively correlated with SWD, SD, and PE, especially with SD. In the soil ecosystem of this study area, the availability or excessive presence of phosphorus exerted a unique impact on the plant community structure [**53**]. For example, excessive phosphorus content may lead to overgrowth of some species that adapt to high phosphorus environments and form dominant species, thereby inhibiting the growth and development of other species and reducing the diversity and evenness of the community [**54**]. In addition, the chemical properties of phosphorus and its transformation process in soil may be different from other elements. Its effect on soil organisms may indirectly affect plant communities by changing soil physical and chemical properties or synergistic effects with other elements, rather than directly affecting species richness as a limiting factor for biological growth [**55**].

MR was significantly positively correlated with SWD, because species richness was an important component of the diversity index. When the species richness increases, the number of different species in the community increases, and the interaction and niche differentiation between species become complex, thereby improving the diversity of the community. The weak positive correlation between the SD and PE suggested that the richness index was not a decisive factor, despite its association with dominance and evenness. In a community with high species richness, a few dominant species may prevail, resulting in an elevated dominance index and diminished evenness index. Conversely, if the species distribution in the community is relatively uniform but the dominant species are not obvious, the community will exhibit a high evenness index and low dominance index.

SWD was positively correlated with SD and PE, indicating a synergistic relationship involving community diversity, the status of dominant species, and the uniformity of species distribution within the plant communities examined in this study. The diversity index reflects the richness and relative abundance of species in the community. The diversity index is elevated when specific dominant species are present in the community, and the species distribution is relatively uniform. The dominant species play an important role in the community, as their resource utilization and ecological functions affect the survival and development of other species, whereas the evenness index reflects the balance of resource allocation among species [**56**]. When the dominant species do not overly monopolize resources and species are evenly distributed, then high diversity is maintained. The positive correlation between SD and PE further indicated that the formation and maintenance of dominant species were related to the uniformity of species distribution in this study area, which may be influenced by the distribution of soil resources and interspecies interactions. This co-variation relationship is crucial for understanding the stability of soil biological community structure and function, because the diversity, dominance, and evenness of the community jointly affect material cycling, energy flow, and ecological service functions of the soil ecosystem.

## 5. Conclusions

In this study, the southern slope of Daxing’anling Forest in Inner Mongolia was used as the research area, and 1a, 3a, 5a, 10a, 20a, abandoned land, and farmland after the restoration were comprehensively selected. The dynamic changes in soil stoichiometric characteristics and plant diversity during the restoration of ecological returning farmland were analyzed, and the effects of soil stoichiometric characteristics on plant diversity were discussed. The conclusions were as follows: (1) In terms of plant communities, plant species initially increased and then decreased after returning farmland, pioneer herbs increased in the early stage, and some species were eliminated due to competition and other factors in the later stage. Shrubs and trees appeared late, and herbs had strong adaptability in all stages. Some plants have wide adaptability and strong transmissibility, which are common in various years, and some plants are harsh to the environment for only a specific year. (2) In terms of soil stoichiometry, the contents of SOC and TN were low in the early stage of returning farmland. With the increase in years, organic carbon increased first and then decreased, and TN increased. The changes of each soil layer were affected by many factors and were different from those of the control. The total phosphorus content fluctuated, and the ratios of C/N, C/P, and N/P had different trends in different soil layers with the conversion of farmland and vegetation restoration. (3) In terms of the relationship between vegetation and soil nutrients, SOC was positively correlated with TN, TN was positively correlated with evenness index, and total phosphorus was negatively correlated with some vegetation indexes. Various positive, negative, and synergistic relationships existed among plant diversity-related indexes, which affect the stability of soil ecosystem. This study comprehensively revealed the evolution of plant community, soil stoichiometric characteristics, and the relationship between vegetation and soil nutrients during the process of returning farmland to forest. These results provide an important scientific basis for further understanding the ecological restoration mechanism and optimizing the strategy of returning farmland to forest. This work helps achieve efficient ecosystem reconstruction and ecological service function improvement.

## Supporting information

S1 File Data. (XLSX)
